# Reproductive effort affects oxidative status and stress in an Antarctic penguin species: An experimental study

**DOI:** 10.1371/journal.pone.0177124

**Published:** 2017-05-11

**Authors:** Roger Colominas-Ciuró, Mercedes Santos, Néstor Coria, Andrés Barbosa

**Affiliations:** 1 Depto. Ecología Evolutiva, Museo Nacional de Ciencias Naturales, CSIC, Calle de José Gutiérrez Abascal 2, Madrid, Spain; 2 Instituto Antártico Argentino–División Biología, Cerrito 1248 (1010), Buenos Aires, Argentina; Centre National de la Recherche Scientifique, FRANCE

## Abstract

The oxidative cost of reproduction has been a matter of debate in recent years presumably because of the lack of proper experimental studies. Based on the hypothesis that different brood sizes produce differential reproductive costs, an experimental manipulation during breeding of Adélie penguins was conducted at Hope Bay, Antarctica, to study oxidative status and stress. We predict that a lower reproductive effort should be positively related to low oxidative and physiological stress. We randomly assigned nests with two chicks to a control reproductive effort group (CRE), and by removing one chick from some nests with two chicks, formed a second, low reproductive effort group (LRE). We examined how oxidative status in blood plasma (reactive oxygen metabolites, ROMs, and total antioxidant capacity, OXY) and stress (heterophil/lymphocyte ratio, H/L) responded to a lower production of offspring total biomass. Our nest manipulation showed significant differences in offspring total biomass, which was lower in the LRE group. As predicted, the LRE group had higher antioxidant capacity than individuals in the CRE group. We have also found, although marginally significant, interactions between sex and treatment in the three variables analysed. Females had higher OXY, lower ROMs and lower H/L ratio when rearing one chick, whereas males did so when rearing two except for OXY which was high regardless of treatment. Moreover, there was a significant negative correlation between the H/L ratio and OXY in females. Finally, we have found a negative and significant relationship between the duration of the experiment and OXY and ROMs and positive with H/L ratio which suggests that indeed breeding penguins are paying an effort in physiological terms in relation to the duration of the chick rearing. In conclusion, a reduction of the reproductive effort decreased oxidative stress in this long-lived bird meaning that a link exists between breeding effort and oxidative stress. However, our findings suggest different sex strategies which results in opposite physiological responses presumably depending on different life-history strategies in males and females.

## Introduction

Identification of proximal causes in the life histories of animal species and their variations, and how traits are associated with stress response are an essential issue in current ecology and evolutionary biology [1]. “Life history theory” is founded on the “Principle of Allocation”, in which there is a trade-off between an increase of resources as energy, nutrients, time, etc. allocated to one function and a corresponding decrease of those invested in another [2]. Thus organisms invest time and energy in reproduction, at the expense of other physiological processes such as flight capability [3], immune system [4] or moult [5] among others, which can result in fitness consequences (e.g. survival rates; [6, 7]). This is commonly known as the cost of reproduction, which is the result of overlapping reproduction, growth and somatic maintenance demands [8]. Such overlap would act as a stressor and might generate a biological response which elicited an individual homeostasis displacement known as stress [9]. For example, egg production is associated with the cost of reproduction in birds [10]. In general, a stronger reproductive effort generates higher reproductive costs, negatively affecting future reproductive attempts and/or self-maintenance ([2, 6, 11], [12] and references therein) by increasing stress [13, 14].

Stress triggers a variety of physiological responses, such as the release of glucocorticosteroids, which is one of the most commonly measured stress markers, although oxidative balance, stress protein responses and leukocyte profile have also been widely examined [14–16].

Biological activity requires energy, and aerobic species use oxygen to release energy efficiently, which produces harmful by-products [17]. Pro-oxidative by-products (reactive oxygen species, ROS) are the result of normal metabolic activities, e.g., aerobic cell respiration [18, 19]. According to the Free Radical Theory [20], free radicals such as ROS, damage some cellular components (e.g. lipids, proteins, and DNA) and their accumulated damage over time contribute to ageing [18, 21]. Biological systems mitigate oxidative damage by means of protection and repair systems, although not necessarily completely efficient ([21] and references therein, [22]). Hence, oxidative stress is defined as the effects of the disruption of normal cell redox signaling on homeostasis [23].The balance between the energy required to counteract the effects of ROS on biomolecules and the potential toxicity of by-products has lately been studied by evolutionary ecologists [24]. Its breakdown might mediate biological functions, such as reproduction, thermoregulation or immune response, among others [1, 25].

The relationship between reproductive effort and oxidative stress has been explored in several animal models by both captive (birds: [26, 27], mammals: [28]) and field [29, 30] experiments. In general terms, and according to Life-History Theory, studies which have tested susceptibility to oxidative stress as a proximal cost of reproduction have found a discrepancy between oxidative status and breeding effort in long and short-lived bird species. In short-lived birds, antioxidant defenses decline with increased reproductive effort [26, 27, 31], whereas long-lived birds would give priority to self-maintenance, increasing their antioxidant capacity while oxidative damage remain unchanged [32]. However, according to [33], there is a lack of clear experimental demonstration on how reproductive effort affects oxidative stress. On one hand due to the absence of an actual manipulated reproductive effort. For instance, using individuals breeding without manipulating the natural brood size (i.e. [32]), or simply manipulating their opportunity to breed (i.e. [28]), may reflect their individual quality or access to resources, even under laboratory conditions, rather than their actual breeding effort [33]. On the other hand, there has been too much focus on antioxidants and not enough on oxidative damage or repair in the experiments carried out (see [26, 27, 29]), and interpretation of results is difficult when damage measurements are lacking [33]. Lately, few experimental demonstrations have filled these gaps. For instance, [34] found differences in sensitivity to oxidative stress in tissues from captive adult female Brandt’s voles (*Lasiopodomys brandtii*, Radde 1861), and although natural reproductive effort suggested an oxidative stress cost with larger litter size, no effect has been found in experimentally manipulated litters. Studies in birds have suggested that rearing enlarge broods depletes plasma non-enzymatic antioxidants in females, but not in males, whereas plasma oxidative damage is unaffected in both sexes [31]. More recently, the combined effect of increased brood size and exposure to ectoparasites was tested for various physiological responses including oxidative stress, and showed that an increase in brood size did affect the antioxidant capacity, but not oxidative damage [30]. However, reproduction costs do not seem to be related to an increase in oxidative damage when conditions are extremely good, as in captivity [35].

Of the various stress measurements, the heterophil/lymphocyte ratio (H/L) is one of the most extensively used [16]. Hematological parameters such as white blood cell counts can also be used to assess stress, because variations in stress hormone levels (glucocorticoids) trigger changes in leukocyte components which can then be quantified (see [16] and references therein). In addition, leukocyte measurements are less sensitive to handling after animal capture than plasma glucocorticoid analyses [16]. In general, the H/L ratio has been found to increase with parental effort ([13], but see [36]). For instance, the long-lived seabird blue-footed boobies showed higher and lower H/L ratio adjustments when their parental reproductive demands were experimentally increased or decreased, respectively [37].

We manipulated the natural Adélie penguin (*Pygoscelis adeliae*, Hombron & Jacquinot 1841) brood size to test whether changes in reproductive effort were associated to costs on stress focusing on changes in oxidative balance (oxidant and antioxidant components) but also on other measures of physiological stress such as H/L ratio by comparing adults rearing one chick or two. Adélie penguins breed in large colonies on land in Antarctica, and must go to the sea for food and return to the colony to feed the chicks. Adélie penguins lay a maximum of two eggs during the austral spring, in October and November, and rear them during the austral summer. Chicks are semi-altricial and nidicolous and are brooded by both parents [38].

Since a higher metabolism may result in the production of more ROS [39, 40], reproduction should trigger susceptibility to oxidative stress unless antioxidant defences also increase to fight off them [26]. Similarly, the heterophil/lymphocyte (H/L) ratio should increase with reproductive effort [13]. Based on the hypothesis that brood size increases reproductive costs, we predict that individuals rearing one chick, and hence, producing less offspring total biomass, should have reduced oxidative damage, increased antioxidant defences, and lower H/L ratio. To our knowledge, this study is one of the few studies linking the cost of reproduction to the oxidative balance in a long-lived bird under natural conditions by means of a direct manipulation of the reproductive effort [35, 41, 42].

## Material & methods

### Ethic statement

This study has been conducted following the Scientific Committee for Antarctic Research Code of Conduct for the use of Animals for Scientific Purposes in Antarctica. The protocol was approved by the Spanish Polar Committee (Permit number CPE-EIA-2012-2).

### Brood size manipulation experiment

The study was conducted in the Adélie penguin rookery at Hope Bay (63°24’S 57°01’W) on the Antarctic Peninsula (Antarctica) during the austral summer of December 2013 to January 2014. Penguins have a maximum clutch size of two eggs; hence, the experimental groups have been formed in accordance to their natural breeding range by reducing brood size in nests with two chicks. Brood size increase in nests with one chick was discarded as we did not know whether clutch size for those were one or two eggs. We selected thirty-three nests on the same day (20/12/2013) with two new born chicks from recently hatched eggs as demonstrated by eggshells around the nest. The nests selected had chicks of similar sizes (t-test: t (1, 49) = -0.476, p = 0.636; mean wing length: 33.74 ± 2.28 mm; min = 29; max = 39). We randomly assigned a low reproductive effort (LRE, one chick) group to even nests and a control reproductive effort (CRE, two chicks) group to odd nests by removing one chick from the LRE group nests. Therefore, the CRE group acts here as a high reproductive effort group compared to LRE. The chick removed was placed in a non-experimental nest which only had one chick of a similar size. After chick manipulation, adults immediately resumed their care. Sample sizes were 17 LRE and 16 CRE nests. No significant differences were found in wing length between LRE and CRE groups (t-test: t (1, 47) = -0.46; p = 0.65; LRE mean wing length = 33.529 ± 2.154 mm; CRE mean wing length = 33.844 ± 2.37 mm).

Around eighteen days (range: 16–23 days, mean = 18.492, SE = 0.203) after selection (20/12/2013) and just before crèching, both adults of each nest were captured (seven adults could not be captured, mean capture date: 07/01/2014 ± 1.5 days, earliest: 05/01/2014, latest: 12/01/2014). Blood samples were taken from a foot vein using a heparinized capillary tube immediately after capture, and one drop was smeared on individually marked microscope slides, air-dried, later fixed in 96% ethanol for 5 minutes and stained with Giemsa pH 7.2 for 30 minutes. The remaining blood sample was later centrifuged at 12000 rpm for 15 min to separate plasma from red blood cells, and both were stored at -20°C until lab processing. In addition, chicks were weighed to the nearest 50 g with a spring balance. At capture, the first adult of a pair sampled was banded for identification. After the second adult was captured, the first adult was recaptured to remove the band. Eighty-nine percent of second adult captures were the day after or two days after the first adult was captured.

### Laboratory analyses

The oxidative balance in blood plasma was measured with both oxidant and antioxidant components using d-reactive oxygen metabolites (d-ROMs) and oxy-adsorbent tests (Diacron International, Grosseto, Italy), respectively. The d-ROMs test measures plasmatic hydroperoxydes, a type of reactive oxygen metabolite (ROM) resulting from oxidative damage (ROS attack on organic substrates such as amino acids, proteins, nucleotides, etc.). The oxy-adsorbent test measures the total plasma antioxidant capacity (OXY), quantifying the contribution of a large section of exogenously and endogenously synthesized antioxidants by adding a highly potent oxidant, hypochlorous acid (HCLO). Then the HCLO radicals which have not reacted can be measured photometrically in a reaction with a chromogenic substrate (see [43] for a similar approach). Both d-ROMs and oxy-adsorbent tests were carried out following [43], with minor modifications, such as using a vortex machine (15 sec.) and short thawed plasma and mixture spins (plasma-reagent, calibrator-reagent and blank-reagent), and incubation with shaking (Speed 6 of 10 in an Amersham Bioscience Hybridization Oven/Shaker) for better homogenization. Absorbance was read at a wavelength of 490 nm with a spectrophotometer at 37°C and shaking (medium speed) the microplate for one minute before reading (Multi-Mode Microplate Reader, Synergy^TM^ HT, BioTek). Measurements were expressed as mg H_2_O_2_ dl^-1^ and μmol HClO ml^-1^ sample for the d-ROMs and oxy-adsorbent tests, respectively. Both d-ROMs and oxy-adsorbent tests were performed using microplates where all treatments were evenly distributed together with duplicates per each sample, blanks and calibrators. Intra-assays coefficients of variation for OXY and d-ROMs for plate I and II were 9.6%, 17.2%, 6.1% and 4.4%, respectively; and inter-assay coefficients of variation for OXY and d-ROMS were 9% and 2%, respectively.

Heterophils and lymphocytes were counted by the same person (RCC) in a total of 100 leukocytes in blood smears under 1000x oil immersion. Leukocytes were counted in the part of the smear where cells were separated in a monolayer to avoid differences in thickness. Then the H/L ratio was calculated (see [44] and references therein for method details). Finally, individuals were sexed by means of molecular markers following [45].

### Statistical analyses

Offspring total biomass (g) per nest that is, the sum of the body mass of the chicks present in the nest, was used as the dependent variable expressing parental effort. Statistical analyses included Student’s t test, simple regressions, one-way ANOVA and general linear mixed models (GLMMs). All variables were normally distributed (Kolmogorov-Smirnov test >0.10). GLMMs were performed on OXY, ROMs and H/L ratio with treatment (LRE and CRE) and sex as fixed factors as well as its interaction, the days of experimentation (days passed between the manipulation date and the sampling date) as a covariate and the pair as a random factor. The mean square (*MS*) and the degrees of freedom (*df*) of the error terms were estimated following the Kenward & Roger method [46]. In addition, the plate used during lab analysis was not added since treatments were evenly distributed among plates. The lack of blood plasma in three samples reduced the OXY and ROMs sample size, therefore, the overall sample size was 58 (25 pairs), 59 (26 pairs), and 59 (26 pairs) individuals for ROMs, OXY and H/L ratio, respectively. All p-values below 0.05 are considered significant. Statistical analyses were performed in the statistical software Statistica 9.0 (StatSoft Inc., USA) and the R packages *lme4* and *lmerTest* [47, 48] run under version 3.3.1 to perform the GLMMs [49].

## Results

Our manipulation of the brood size to find any differential parental effort in the two experimental groups (LRE and CRE) resulted in differences in the offspring total biomass (g) per nest (t = -9.73; p < 0.001, n_LRE_ = 17, n_CRE_ = 16; LRE mean = 1575 ± 281.597; CRE mean = 2753 ± 406.702). This means that the individuals included in the LRE group had worked less than those in CRE. However, CRE penguins did not work twice as much as the LRE ones.

Our results did not show significant differences between treatments and between sexes in relation to the days of experimentation (treatment: F(1,58) = 0.706, p = 0.404208, LRE mean: 18.62500 ± 0.294881 and CRE mean: 18.28571 ± 0.294881; and sex: F(1,57) = 0.290, p = 0.592343, male mean: 18.60714 ± 0.296210 and female mean: 18.38710 ± 0.281512).

**Fig 1:**
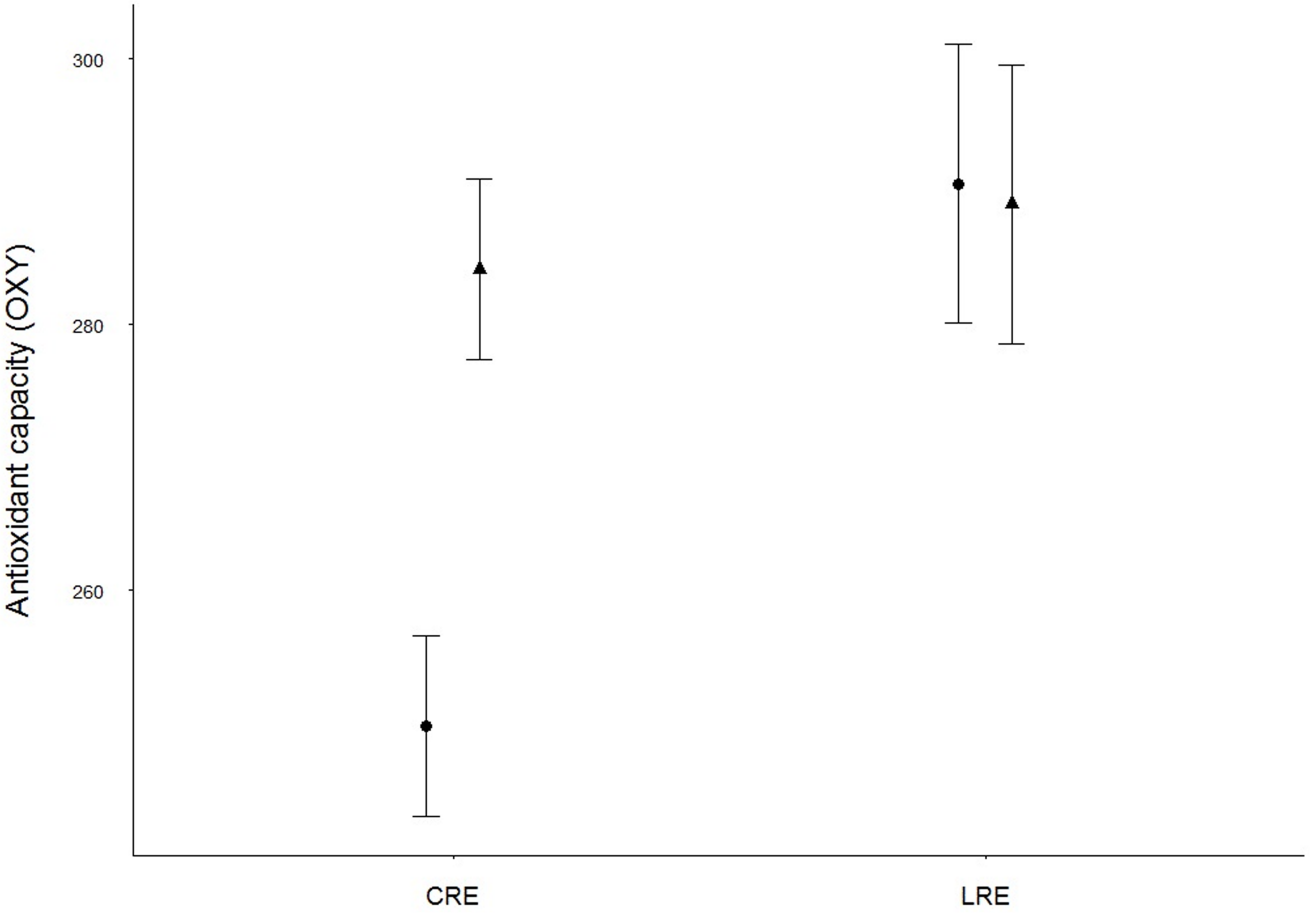
Antioxidant capacity (OXY, μmol HClO ml^-1^) differences between treatments and sexes (males: filled triangles; females: filled circles) in Adélie penguins. Values are shown as means ± s.e.

**Fig 2:**
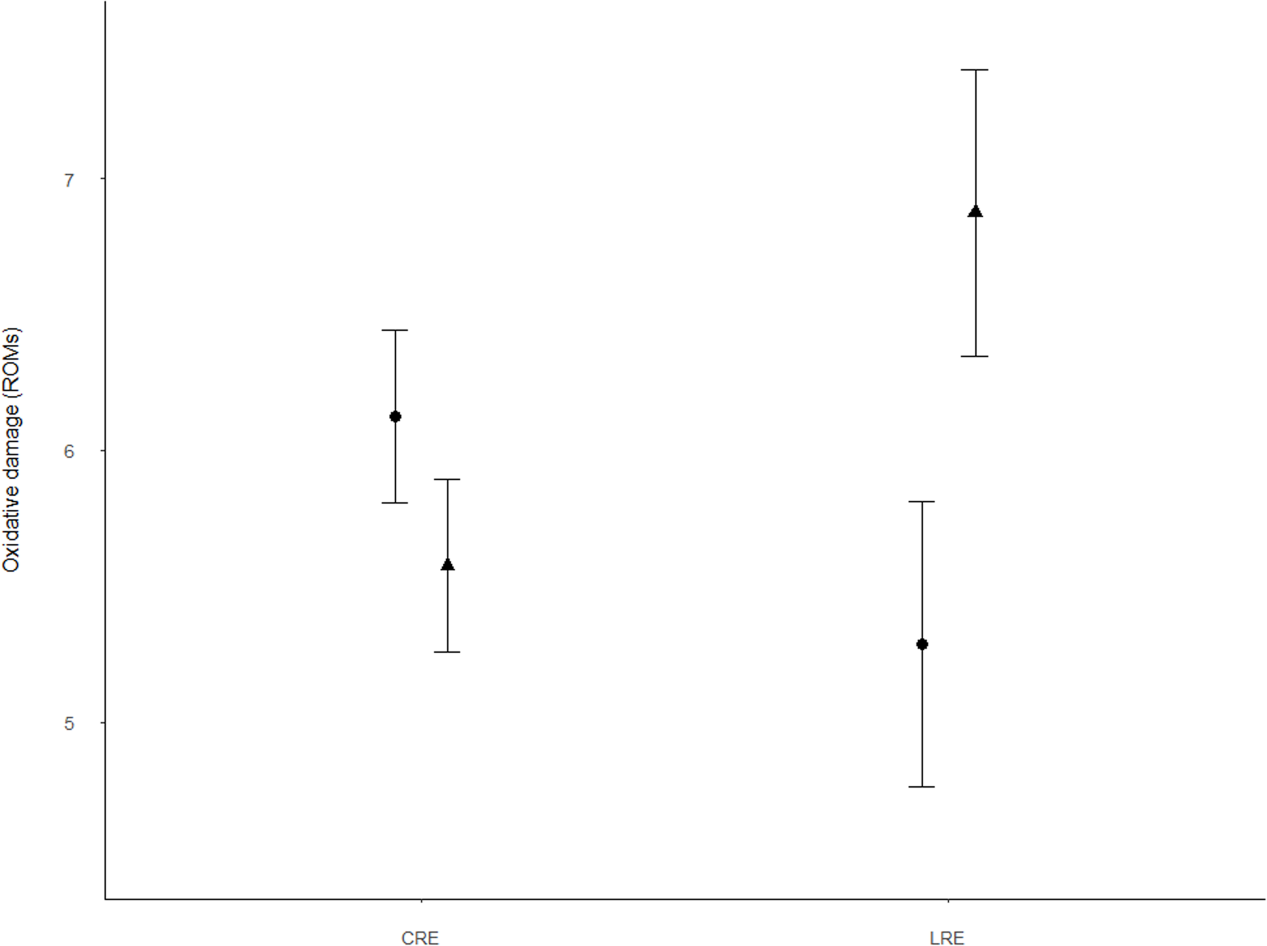
Oxidative damage (ROMs, mg H_2_O_2_ dl^-1^) differences between treatments and sexes (males: filled triangles; females: filled circles) in Adélie penguins. Values are shown as means ± s.e.

**Fig 3:**
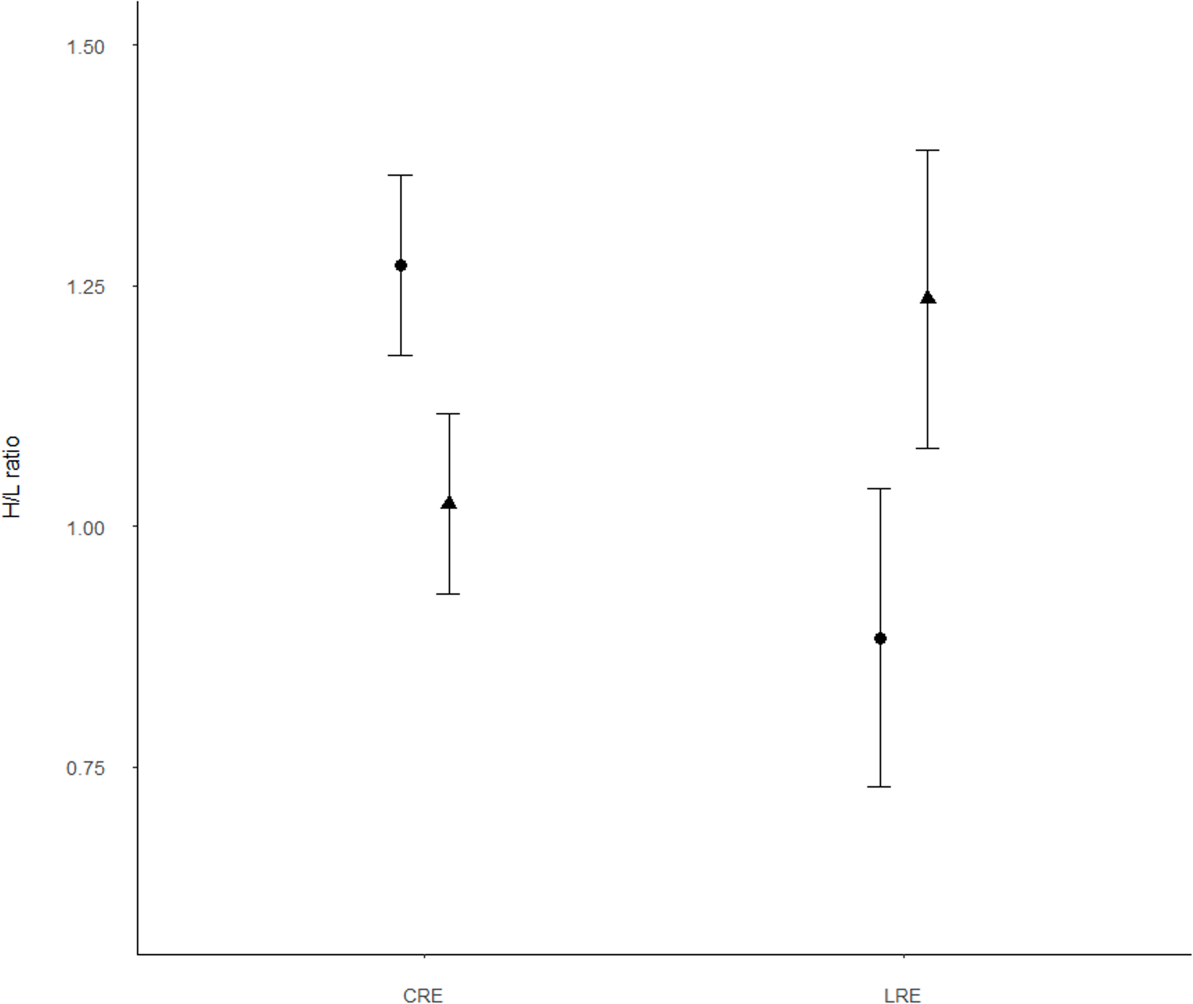
Heterophil/lymphocyte ratio differences between treatments and sexes (males: filled triangles; females: filled circles) in Adélie penguins. Values are shown as means ± s.e.

**Fig 4:**
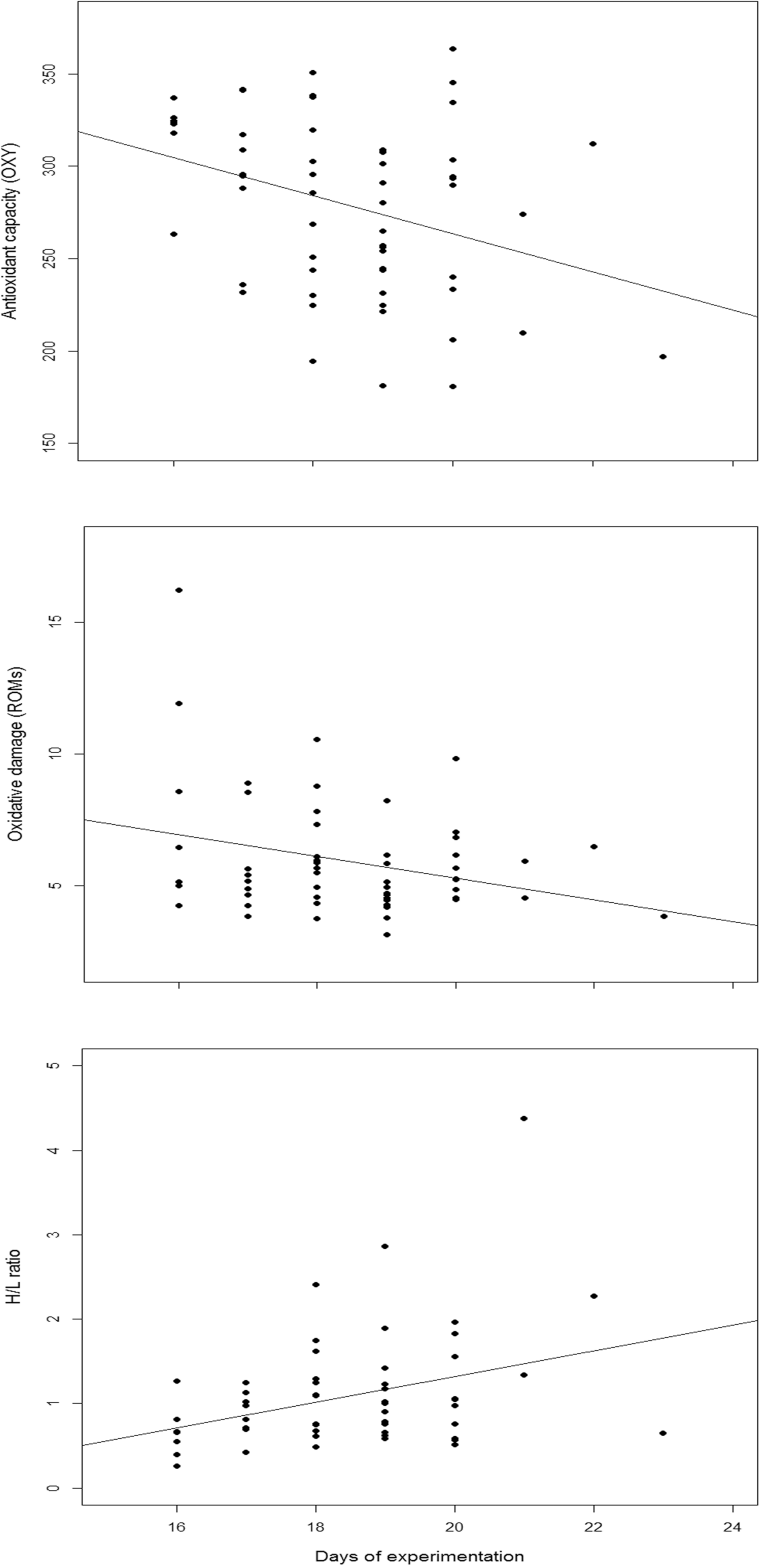
Relationships between the duration of the experiment (days of experimentation) and the antioxidant levels (OXY), the oxidative damage values (ROMs) and the H/L ratio.

**Fig 5:**
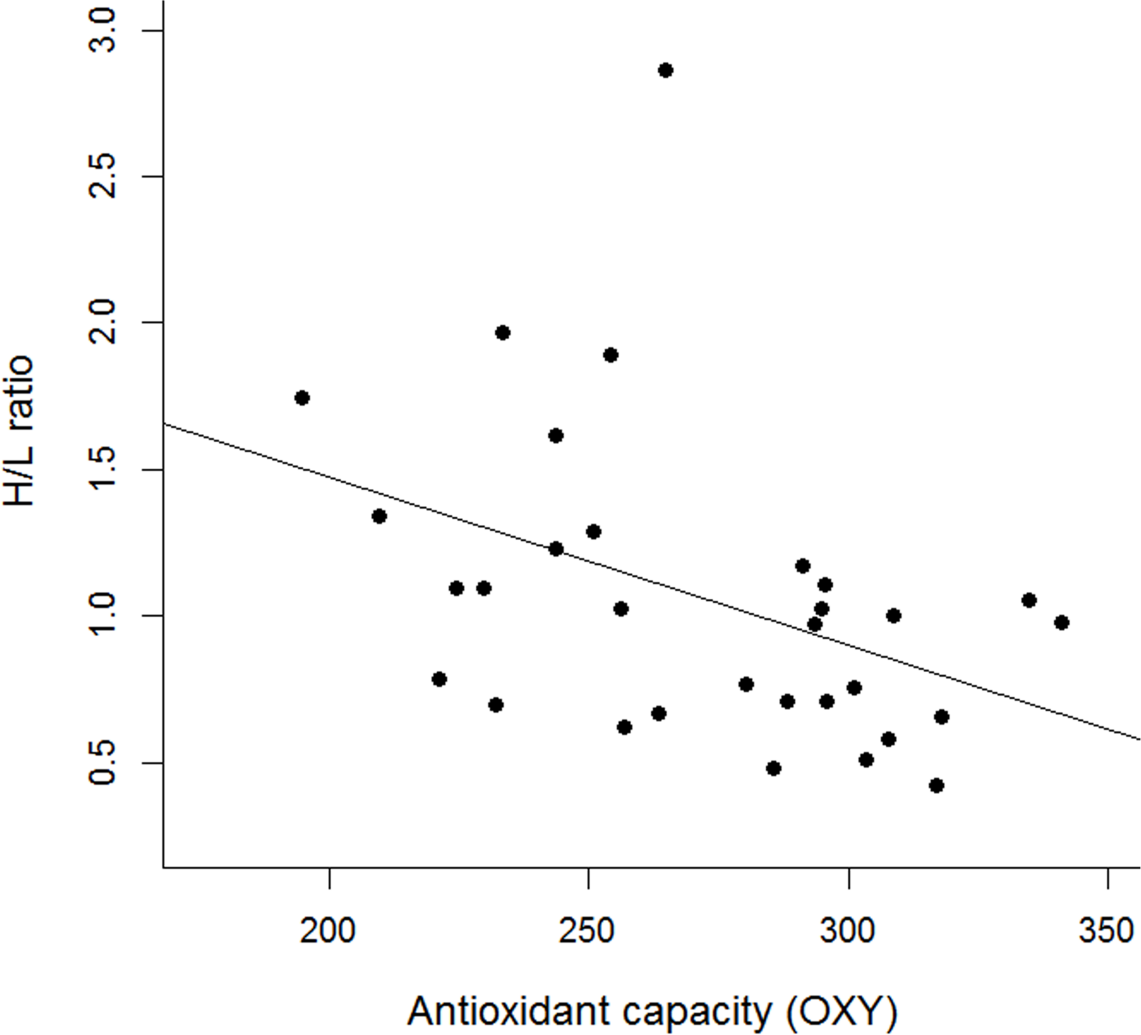
Relationship between the antioxidant capacity (OXY, μmol HClO ml^-1^) and the heterophil/lymphocyte ratio in Adélie penguin females.

Results from the GLMM showed that adults in the LRE group had significantly higher antioxidant levels (OXY) than those in CRE (Tables 1 and 2, Fig 1). This was because females in the LRE group had higher antioxidant levels than those in CRE although marginally significant (Tables 1 and 2, Fig 1). In contrast, treatments did not have any effects in males (Fig 1). Significant differences in antioxidant capacity by sex were also marginally significant regardless of treatment (Tables 1 and 2). Furthermore, the response of adults regarding oxidative damage (ROMs) and H/L ratio was not significant for treatment or sex, although tended to diverge in its interaction: females tended to show lower values when brood size was reduced, while those in males tended to be higher (Table 2, Figs 2 and 3). GLMM results also showed a significant negative relationship between the duration of the experiment (days of experimentation) and the antioxidant levels (OXY) and oxidative damage (ROMs) and a significant positive relationship with the H/L ratio (Table 2, Fig 4). Simple regressions showed a significant negative relationship between H/L ratio and antioxidant capacity (OXY; r = -0.42, p = 0.02, n = 31; Fig 5) in females, but not in males (r = - 0.03, p = 0.9, n = 28). Finally, no significant relationships were found between H/L ratio and oxidative damage for either sex (Females: r = 0.22, p = 0.24, n = 31; Males: r = - 0.15, p = 0.46, n = 27).

**Table 1:** Mean values of the response variables related to the fixed factors analyzed.

		OXY	ROMs	H/L ratio
Sex	Male	289.384 ± 7.168	6.257 ± 0.427	1.129 ± 0.127
	Female	268.698 ± 6.853	5.709 ± 0.402	1.078 ± 0.121
Treatment	LRE	289.421 ± 6.814	6.076 ± 0.4	1.06 ± 0.121
	CRE	268.661 ± 7.205	5.89 ± 0.429	1.147 ± 0.127

Values are means ± standard error.

**Table 2:** Results of a general mixed ANCOVA GLMM, examining the effects of the sex, the treatment and the days of experimentation of Adélie penguins on oxidative status and H/L ratio. Denominator degrees of freedom follow the Kenward-Roger approach to GLMM. For more details see the Methods section.

	OXY				ROMs				H/L ratio			
	Estimate	DF	F	p	Estimate	DF	F	p	Estimate	DF	F	p
Sex	-9.771	1,27.886	3.624	0.067	-0.299	1,28.373	1.101	0.303	-0.007	1,28.542	0.006	0.938
Treatment	-13.950	1,29.504	5.760	0.023*	-0.218	1,29.237	0.571	0.456	0.076	1,29.071	0.823	0.372
Sex x Treatment	-9.705	1,28.004	3.596	0.068	0.537	1,28.385	3.557	0.07	0.154	1,28.599	3.467	0.073
Days of experimentation	-11.637	1,52.454	9.789	0.003*	-0.444	1,48.800	5.365	0.025*	0.159	1,49.303	8.258	0.006*

* Indicate significant difference if P<0.05.

## Discussion

Demanding situations as reproduction may increase metabolism and might produce more ROS, unless antioxidant defences also increase to fight off them [26]. Our results are consistent with this notion, first, we found that the duration of the experiment (i.e.: days of experimentation) was positively related with stress (lower OXY and higher H/L, although lower ROM) suggesting that breeding penguins are paying an effort in physiological terms including oxidative balance in relation to the duration of the chick rearing. However, more research based on longitudinal data would be needed to confirm this result.

Second, our results show that females of Adélie penguins in the LRE group rearing one chick instead of two had higher antioxidant defences than individuals in the CRE group rearing two chicks which partially support our prediction. Therefore, our study suggests that the female Adélie penguins currently experiencing different breeding efforts respond changing their physiological status in terms of oxidative balance. This study experimentally demonstrates under natural conditions, a direct effect of the reproductive effort on the oxidative balance in a long-lived bird. The relationship between reproductive effort and oxidative stress has been studied in several animal models by both captive (birds: [26, 27], mammals: [28]) and field [29, 30] experiments. In general, these authors found that the increase in the breeding effort is linked with a decrease of defences against ROS [26, 27, 31] or a decrease in the resistance of oxidative stress [29]. However, some authors have found contradictory results with a higher antioxidant capacity in a high breeding effort situation [30] and some others have not found any relationship between breeding effort and oxidative damage [34, 50]. Overall, our results are in accordance with those where a reduction of brood size increases the antioxidant barrier in birds [26, 27]. Recently, the “oxidative shielding theory” proposes that females could have been selected to diminish oxidative damage which would explain the lack of clear results showing an oxidative cost of reproduction for mothers [51]. However, our results show that Adélie penguin females could have reproduction costs expressed in the reduction of antioxidant defences in contrast with that of the hypothesis proposes, although we found no oxidative costs in males.

Oxidative stress and breeding effort has been previously studied in the Adélie penguin [32] through a manipulation of the locomotion performance during the breeding period possibly impacting on reproductive success [31], although cannot be considered strictly as an actual manipulation of the breeding effort (see [33]). The results found by [32] showed an increase in the antioxidant defences during breeding in the handicapped individuals with reduced hydrodynamic properties, that is, those with the higher locomotion effort. Although the experimental performance carried out by [32] and the present study disentangle different approaches to study breeding efforts by using different kind of manipulations, decreasing (the present study) or increasing [32] the reproductive effort, the results in both studies show that a variation of the effort carried out by the penguins increases antioxidant defenses whereas oxidative damage remains stable. This suggests the existence of a U-curve reflecting the relationship between reproductive effort and antioxidant defences. Adélie penguins seem to set their reproductive effort to a value allowing them to mobilize the lowest levels of antioxidant defences. However, a mismatch between this initial value of reproductive effort and the actual reproductive effort, which are triggered by the experimental manipulations, results in increased antioxidant defences. More research is needed to confirm this suggestion.

Our results also show, although marginally significant, that changes in the oxidative balance seem to diverge between females and males. Whereas males tend to show more stress in terms of high oxidative damage and H/L ratio when rearing one chick, females tend to show the opposite, undergoing more stress when rearing two chicks. Further, females suffered more oxidative stress than males when rearing two chicks since antioxidant defences were lower. Based on our results, rearing chicks appears to be a more demanding activity for females than for males. Sex differences in oxidative balance have previously been reported for both short-lived [26, 27, 30] and long-lived captive [35] species, showing conflicting results. However, sex differences presented here have never been previously found in Adélie penguins. For example, in captive zebra finches (*Taeniopygia guttata*, Vieillot 1817), antioxidant defenses appeared to decrease more in males than females with increased brood size [26]. Male and female captive canaries responded similarly when brood size was manipulated, however, females tended to have more protein damage and a higher concentration of non-protein thiols, that is, a higher antioxidant defence, than males [35]. Our results may be explained by three non-exclusive scenarios: (i) Different initial breeding investment between sexes, (ii) Sex-bias in foraging effort and spatial distribution at sea, and (iii) Sex-bias of parental defence of the nest.

(i) Considering that metabolic costs of courtship behaviour are low, that incubation has little or no metabolic cost in Adélie penguins [52], that the males probably produce sperm at small cost [53], and that the cost to females of egg or gametes production is very low due to the small clutch size, this explanation can be ruled out. (ii) Adélie female foraging trip distances and duration have been reported to be significantly greater than those by males during the guard phase [43, 54–56] and this can be dependent of the sex of the chicks showing more flexibility of resource allocation in females [57]. Therefore, even though an equal feeding rate seems to be shared by both parents [58], sex-bias foraging at sea may explain our results. (iii) Differential nest defence by the two sexes could also explain sex differences in stress. Males show stronger agonistic interaction than females in other closely related species, such as the chinstrap penguin (*Pygoscelis antarcticus*, Forster 1781; [59]), which might increase glucocorticoids [16] due to social stressors, such as nest defence and stone-collecting behaviour, but see [60]. A larger number of agonistic interactions might indicate a higher metabolism, which could trigger more ROS production, resulting in greater oxidative damage [26]. In addition, it is therefore likely that sex differences in terms of H/L ratio are also related to a wider difference in the number of interactions between males and females [61]. Differences found within males rearing one or two chicks could be explained because males with one chick may increase nest defence to ensure survival of the single chick (see [62] for an evidence of nest defence intensity to be related to changes in the reproductive value of the brood).

Our results could also be explained by differential investment by males and females in parental care. However, Adélie chicks are known to be brooded by both parents, who exchange duties every day or so [63–65], and this behaviour was also recorded in our penguin rookery [58]. Unfortunately, we have no data on nest attendance in this study, although we recaptured 89% of second adults within 1–2 days after the first adult which is coincident with reliefs recorded by [58], and therefore, we can rule out this explanation as responsible for our results.

We also found a significant negative correlation between the female H/L ratio and their antioxidant capacity showing that antioxidant defences are lower in individuals with high stress. Stress factors trigger several physiological responses, such as the release of glucocorticosteroids which result in quantifiable changes in leukocyte components [16], or an increase in ROS production that could damage biomolecules without antioxidant action [17, 19]. As oxidative stress is involved in infectious and/or inflammatory processes [22, 66], negative relationships between leukocyte markers, such as the H/L ratio and antioxidant capacity, would be expected (see [67]). In fact, these authors found a negative non-linear correlation between total antioxidant capacity and both absolute and relative concentration of heterophils and H/L ratio in great tits (*Parus major*, Linnaeus 1758). Such negative relationships between antioxidant defences and the H/L ratio have also been found in birds in an immune challenge ([68], e.g.: PHA injection; [69]). In our case, we found such relationships only in females, which reinforces the idea that in Adélie penguins, females are more affected than males by breeding stress, as is also reflected at the level of oxidative balance.

In conclusion, our results suggest that reproductive effort increases oxidative stress and susceptibility to stress in a long-lived bird like the Adélie penguin and seem to suggest that both sexes could have different strategies for coping with increased reproductive costs, which results in opposite physiological responses depending on how each sex performs life-history traits. However, more research is needed to confirm such results.
